# The characteristics of perivascular proinflammation in 4‐repeat tauopathies

**DOI:** 10.1111/bpa.70162

**Published:** 2026-10-28

**Authors:** Mu‐Hui Fu, Gokhan Uruk, Rodolfo G. Gatto, R. Ross Reichard, Jennifer L. Whitwell, Keith A. Josephs

**Affiliations:** ^1^ Department of Neurology Mayo Clinic Rochester Minnesota USA; ^2^ Department of Neurology Kaohsiung Chang Gung Memorial Hospital and Chang Gung University College of Medicine Kaohsiung Taiwan; ^3^ Department of Laboratory Medicine and Pathology (Neuropathology) Mayo Clinic Rochester Minnesota USA; ^4^ Department of Radiology Mayo Clinic Rochester Minnesota USA

**Keywords:** arteriole, corticobasal degeneration, neuroinflammation, perivascular proinflammation, progressive supranuclear palsy, venule

## Abstract

Progressive supranuclear palsy (PSP) and corticobasal degeneration (CBD) are both neurodegenerative diseases characterized by aggregates composed of 4‐repeat (4R) tau protein. They have distinct neuropathological features and classic clinical symptoms; however, the signs and symptoms overlap across the two syndromes. Accumulating evidence suggests that dysregulated immune response is a major contributing factor to neurodegeneration but its role in 4R‐tauopathies remains undetermined. The aim is to explore the involvement of neuroinflammation from a vascular perspective in 4R‐tauopathies. Autopsies from four controls, three PSP, and three CBD cases were assessed. Samples were obtained from the superior/middle frontal gyrus (S/M‐FG) and basal ganglia (BG). Immunofluorescence staining included neuroinflammatory markers: glial fibrillary acidic protein (GFAP), ionized calcium‐binding adapter molecule 1 (Iba1), cluster of differentiation 68 (CD68); vascular markers: collagen type IV (ColIV), monocarboxylate transporter‐1 (MCT1), and α‐smooth muscle actin (*α*SMA). Maximally projected confocal z‐stack images of vasculature were used as a mask to quantify total perivascular GFAP+/Iba1+/CD68+ labeling. GFAP immunoreactivity ratio between MCT1 and *α*SMA double labeled arteries and veins was quantified to further differentiate venular and arteriolar involvement into proinflammatory activation. Both PSP and CBD had widespread perivascular proinflammatory changes, although greater in CBD. The activated GFAP+ astrocytes and microglia/macrophages in CBD were distributed extensively in S/M‐FG and BG, with S/M‐FG WM more affected. In PSP, abundant neuroinflammation was found in BG. The involvement of vasculature type was distinct among the two 4R‐tauopathies, while CBD showed comparable proinflammatory changes in arterioles and venules, PSP exhibited predominant proinflammation in venules. The regional pattern of perivascular proinflammation differed between PSP and CBD, corresponding to the characteristic affected brain regions. CBD had more severe perivascular proinflammation that involved both arterioles and venules, while venules were more affected in PSP. Perivascular proinflammation may be a critical pathophysiological process in 4R‐tauopathies.

## Graphical abstract

The spatial distribution of perivascular proinflammation differs between PSP and CBD: PSP shows a predominance in basal ganglia (BG), whereas CBD demonstrates more widespread involvement of both BG and superior/middle frontal gyrus (S/M‐FG). PSP exhibits profound proinflammatory activation around venules while CBD affects a comparable magnitude around both arterioles and venules.

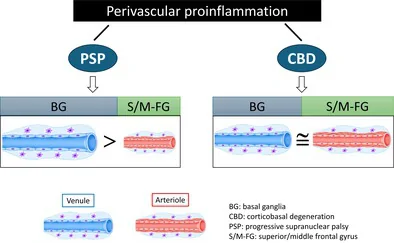

## 1 INTRODUCTION

Progressive supranuclear palsy (PSP) and corticobasal degeneration (CBD) are neurodegenerative disorders which are both defined by deposition of 4‐repeat (4R) tau. PSP has tau pathology in the neurons of the pallido‐nigro‐luysian axis with the characteristic feature of midbrain atrophy [1], while CBD has more cortical neuronal and glial tau pathology affecting parasagittal superior frontoparietal regions asymmetrically [2]. Although CBD can be differentiated from PSP pathologically, mixed clinical, radiological, and pathological pictures have been reported [3, 4, 5]. The classic clinical phenotype associated with CBD is known as corticobasal syndrome (CBS) and is characterized by a variety of cortical signs like alien hand syndrome, ideomotor apraxia, cortical sensory deficits, and asymmetric extrapyramidal symptoms including parkinsonism, dystonia, and myoclonus [6, 7]. The classic phenotype associated with PSP is characterized by postural instability with early falls, supranuclear vertical gaze palsy, and subcortical frontal dementia [8]. However, overlap is often observed, with CBS resulting from PSP pathology, and vice versa [9, 10], making clinical diagnosis challenging.

There is growing evidence indicating an important role of neuroinflammation in neurodegenerative disease [11, 12]. It is widely accepted that inflammation is a standard and common response to disease‐associated misfolded proteins, including tau [13, 14, 15]. Microglia are the major resident immune cells of the CNS, and mounting data support their importance in the neurodegenerative diseases [16]. In tauopathies, reactive microglia co‐localize with tau oligomers, clustering in the vicinity of neurofibrillary tangles and corresponding well with the distribution of neuropathological change in Alzheimer's disease (AD), PSP, and CBD [17, 18, 19]. Rather than being a consequence, recent studies suggest that inflammation develops in an even earlier stage or as an initiator of neurodegeneration. Factors like traumatic brain injury [20], systemic inflammation [21], obesity [22], aging [23], dysbiotic gut microbiota [24], and cerebrovascular impairment [25] were reported as stimulants of neuroinflammation. In a P301S tauopathy mouse model, microglia activation developed prior to tau aggregation [26]. Besides, elevated levels of proinflammatory cytokines have been observed in the cerebrospinal fluid (CSF) of patients with mild cognitive impairment [27]. Despite being linked to several neurodegenerative diseases, the role of neuroinflammation in 4R‐tauopathies has not been elucidated.

Central nervous system (CNS) has been regarded as an immune‐privileged region for the existence of a specialized border‐blood brain barrier (BBB), to prevent brain from the invasion of peripheral blood toxins or pathogens [28]. Disruption of BBB is an early sign of neurodegenerative diseases and plays crucial role in their pathomechanism [25]. Among the BBB neurovascular unit, astrocyte is the major glial cell maintaining BBB function and is represented by a marker—GFAP [29]. The GFAP levels are highly upregulated during neuroinflammation and neurodegeneration because of astrogliosis [30, 31]. The crosstalk between neuroinflammation and neurodegeneration is sprouting but the related studies on neuroinflammation in 4R‐tauopathies is limited.

Another major component of the BBB is the specialized microvascular endothelial cells. While responding to systemic inflammation, the dysfunctional microvascular endothelial cells express receptors for cytokines and facilitate peripheral immune cells entering the brain through the disrupted BBB. White matter hyperintensities (WMHs) are a marker of cerebral small vessel diseases presumed to correlate with venous collagenosis or penetrating arterial occlusion [32, 33], and increased WMHs have been observed in AD and PSP patients [34, 35, 36]. While vasculopathy has been extensively studied in AD [37], it remains relatively underexplored in 4R‐tauopathies.

The aim of this study is to explore the pattern of neuroinflammation from a vascular perspective in two 4R tauopathies, PSP and CBD. Microscopic abnormalities in the basal ganglia (BG), diencephalon, and brainstem are the cardinal findings of PSP pathology [38], while asymmetric frontoparietal atrophy is a key imaging picture of CBD [39]. Our prior study suggested that the distribution of WMHs in the subcortical variant of PSP complied with the territory of the deep venous system [40]. Frontal gyrus and BG are within different drainage territories of two cerebral venous systems—superficial and deep; thus, the two regions were chosen to delineate the inflammatory change around vascular structures. We hypothesize that the distribution of perivascular inflammation is distinct and relevant to the brain regions vulnerable to the pathology of the two 4R tauopathies.

## 2 MATERIALS AND METHODS

### 2.1 Study subjects

Between September 2009 and October 2023, we prospectively recruited participants with atypical Parkinsonian syndromes that were referred to and enrolled in one of several NIH‐funded studies conducted by the Neurodegenerative Research Group (NRG) at Mayo Clinic, Rochester, Minnesota. All participants were evaluated by a movement disorder and behavioral neurology specialist (KAJ). For this study, we selected three patients with autopsy confirmed PSP and three patients with autopsy confirmed CBD (Table S1). We also identified four patients who died without neurodegenerative pathology (i.e., Braak stage = 0, Thal phase = 0) to act as controls (Table S1).

Informed consent was obtained from all subjects for participation in the study, which was approved by the Mayo Institutional Review Board.

### 2.2 MRI imaging

All participants had undergone an antemortem 3T MRI protocol on a GE scanner that included a magnetization‐prepared rapid gradient‐echo (MPRAGE) sequence (TR/TE/T1 = 2300/3/900 ms; 26‐cm FOV; slice thickness = 1.2 mm; in‐plane resolution = 1 mm). An additional fluid‐attenuated inversion recovery (FLAIR) sequence was acquired with a relaxation and excitation time (TR/TE) of 10,000/125.05 ms, fat suppression (FS) equal to 3, field of view of 22‐cm, and a slice thickness of 5 mm.

### 2.3 Multiplex immunofluorescence studies

For each case, a total of five specific brain regions‐of‐interest (ROIs) from two formalin fixed paraffin‐embedded (FFPE) human brain tissue blocks spanning frontal gyri and BG were selected for analysis and neuropathological examination: (1) white matter (WM), (2) gray matter (GM) regions of superior‐middle frontal gyrus (S/M‐FG), (3) Globus pallidus‐internus (GPi), (4) Globus pallidus‐externus (GPe), and (5) Putamen (Put) of BG. Block sections were cut into 10 μm. The sections were permeabilized with 0.3% Triton‐X 100‐1X phosphate buffered saline (PBS) at room temperature for 30 min, then antigen retrieval was performed with 10 mM sodium citrate (pH: 6.0) at 80°C for 30 min. The sections were incubated with unconjugated AffiniPure™ F(ab′)_2_ Fragment Donkey Anti‐Human IgG (H + L) (Jackson Immunoresearch, catalog # 709‐006‐149), diluted 1:35 in 1X PBS overnight at 4°C. On the following day, the sections were incubated in blocking buffer (2% normal donkey serum and 0.1% bovine serum albumin (BSA) in 1X PBS) for 1 h, and then incubated with primary antibodies overnight at 4°C. After washing, the sections were incubated with secondary antibodies for 2 h (Jackson Immunoresearch, 1:200 in 1X PBS). Samples were then incubated with TrueBlack® lipofuscin autofluorescence quencher (Biotium, catalog #23007) for 3 min at room temperature and then washed in 1X PBS for 5 min 3 times. Stained sections were mounted on glass slides in antifade mounting medium (Vector laboratories, VECTASHIELD Vibrance® Antifade Mounting Medium (H‐1700)). Control sections omitting either primary or secondary antibodies showed no signal above background.

To assess the microglia/macrophages, astrocytes, and vasculature across the cases, primary antibodies were obtained from the following sources and used at various dilutions (1) ionized calcium‐binding adapter molecule 1 (Iba1) for microglia and macrophages (Rabbit polyclonal, Fuji film, Cat # 019‐19741, 1:400), (2) Iba1 (Guinea pig monoclonal, Synaptic Systems, Cat # HS‐234308, 1:200), (3) cluster of differentiation 68 (CD68) for lysosomal glycoprotein being upregulated during activation (Rat Monoclonal, BioRad, Cat # MCA1957, 1:400), (4) GFAP (Chicken polyclonal, Abcam, Cat # ab4674, 1:400), (5) Collagen Type IV (ColIV) (Goat polyclonal, Abcam, Cat # AB769, 1:400). The staining of Iba1 and CD68 reflects the proinflammatory response of microglia/macrophage.

To further characterize the relationship between vascular compartments and pro‐inflammatory activation, we labeled venules with (6) Monocarboxylate transporter‐1 (MCT1; Rabbit polyclonal, Proteintech, Cat #20139‐1‐AP, 1:200) and arterioles with (7) Anti‐Actin, *α*‐Smooth Muscle–Cy3™ (αSMA; Sigma‐Aldrich, Mouse monoclonal‐conjugated, Cat #C6198, 1:200), together with GFAP.

To identify the spatial relationship between perivascular proinflammatory activation and tau accumulation, we labeled tau with (8) anti‐AT8 phosphorylated tau (Thermo Fisher Scientific, catalog #MN1020, 1:200), which detects pre‐stable tangles. Vasculature and astrocytes/microglia were labeled as previously explained.

Secondary antibodies were obtained from the following sources and used at 1:200 dilutions: Alexa Fluor 488 Donkey Anti‐Rabbit IgG (H + L) (Jackson Immunoresearch, Cat # 711‐545‐152), Alexa Fluor® 405 AffiniPure™ Donkey Anti‐Chicken IgY (IgG) (H + L) (Jackson Immunoresearch, Cat # 703‐475‐155), Alexa Fluor® 647 AffiniPure™ Donkey Anti‐Chicken IgY (IgG) (H + L) (Jackson Immunoresearch, Cat # 703‐605‐155), DyLight™ 405 AffiniPure™ Donkey Anti‐Guinea Pig IgG (H + L) (Jackson Immunoresearch, Cat # 706‐475‐148), DyLight™ 405 AffiniPure™ Donkey Anti‐Rat IgG (H + L) (Jackson Immunoresearch, Cat # 712‐475‐153), Alexa Fluor 568 AffiniPure™ Donkey Anti‐Rat IgG (H + L) (Jackson Immunoresearch, Cat # 712‐575‐153), Alexa Fluor® 647 AffiniPure™ Donkey Anti‐Goat IgG (H + L) (Jackson Immunoresearch, Cat # 705‐605‐147).

### 2.4 Detection and quantification of astrocyte and microglia/macrophage morphological responses

All multiplex images were prepared with a spinning disc Zeiss 980 LSM confocal microscope under identical laser illumination and capture conditions. Both the people taking and analyzing the images were blinded to the treatment conditions. For brain sections, confocal images were taken at three locations that were a minimum of 100 μm apart from each other under 20× magnification. The number of images taken from each region was dependent on the size of the spanned area, which was significantly affected by disease processes. The confocal images were *z*‐stacked, and each processed image was prepared using a stack of 10 1‐μm thick Z‐stage images. The total number of microglia/macrophage and astrocyte responses was assessed [41, 42]. The total integrated density of GFAP+/Iba1+/CD68+ cells was assessed using a custom‐made ImageJ/Fiji macro by being thresholded using the Triangle function and size exclusion. The masks created in this process were also used to quantify Iba1+/CD68+ microglia, and GFAP+ astrocyte extension area was quantified after excluding the cell body areas on ImageJ using the particle size function.

### 2.5 Perivascular astrocyte and microglia/macrophage assessments

Vascular tree, venules, and arterioles were labeled with ColIV, MCT1, and *α*SMA, respectively [43]. Astrocytes were tagged with GFAP, while Iba1 was used to detect microglia/macrophages. Anti‐CD68 antibody was performed to label activated microglia/macrophages. The *z*‐stack images were maximally projected, and images depicting vasculature were used as a mask via custom‐made ImageJ/Fiji macro by being thresholded using the Otsu function [44]. Total number of GFAP+/Iba1+/CD68+ area coverage surrounding the vessels was quantified. The immunoreactivity ratio around the venular and arteriolar system was quantified by dividing the integrated density values.

### 2.6 Statistical analysis

The “*n*” of each study defined as the number of independent experiments from each case. Numerical data were expressed as means ± SEM, analyzed using one‐way ANOVA by either the Tukey–Kramer test or Dunnett's test where multiple groups were compared to each other or against a common control group. Where only two groups were compared, the two‐sided *t*‐test was used. All data analysis was performed by individuals who were blinded to the experimental conditions. *p*< 0.05 was considered statistically significant.

## 3 RESULTS

### 3.1 Antemortem imaging

Representative MRI FLAIR images are shown in Supplementary Figure 1. WMHs were observed surrounding the ventricles in both the representative PSP and CBD cases with more frontal involvement in CBD (Figure S1A, line and arrow), and hypointensity was observed in the Put and GP subregions in both PSP and CBD cases (Figure S1B). The pathological specimens were taken from the frontal gyrus and BG, the regions close to the WMH‐present areas.

### 3.2 Perivascular proinflammatory activation and changes in PSP and CBD

Immunofluorescence images of Iba1, CD68, GFAP and Collagen IV, and overlap images, in the WM of the S/M‐FG in representative cases are shown in Figure 1A, with quantitative total integrated density shown in Figure 1B–D. Perivascular GFAP+ density (i.e., density around the vessels) was greater in CBD than PSP, and greater in PSP than controls (Figure 1A,B). For Iba‐1+, only CBD demonstrated higher density than controls (Figure 1A,C). For CD68+, CBD showed greater density than controls and PSP (Figure 1A,D). In the GM of the S/M‐FG, increased reactive astrocytosis was observed in both PSP and CBD, although no difference was observed between PSP and CBD (Figure 2A,B). No group differences were observed in the perivascular Iba1+ density (Figure 2C). However, the perivascular CD68 density was greater in PSP and CBD compared to controls, and greater in CBD compared to PSP (Figure 2D). Also, comparing WM and GM regions of the S/M‐FG demonstrated that the WM seemed to be affected greater than the GM in CBD due to burden of each marker: however, perivascular GFAP/ Iba1/ CD68 intensity analyses revealed no difference among WM and GM (Figures 1 and 2, S4). In the GPi (Figure 3A), both PSP and CBD showed greater perivascular GFAP+ and CD68+ density than controls, with no differences observed between PSP and CBD (Figure 3B–D). The GFAP+ findings were similar in the GPe and Put, although CD68+ density only differed between CBD and controls in GPe (Figures S2 and S3). It is interesting to note that GPi had the greatest proinflammatory activation morphologically among the other BG regions; however, perivascular intensity analyses failed to reveal this pattern (Figures 3 and S2–S4).

**FIGURE 1:**
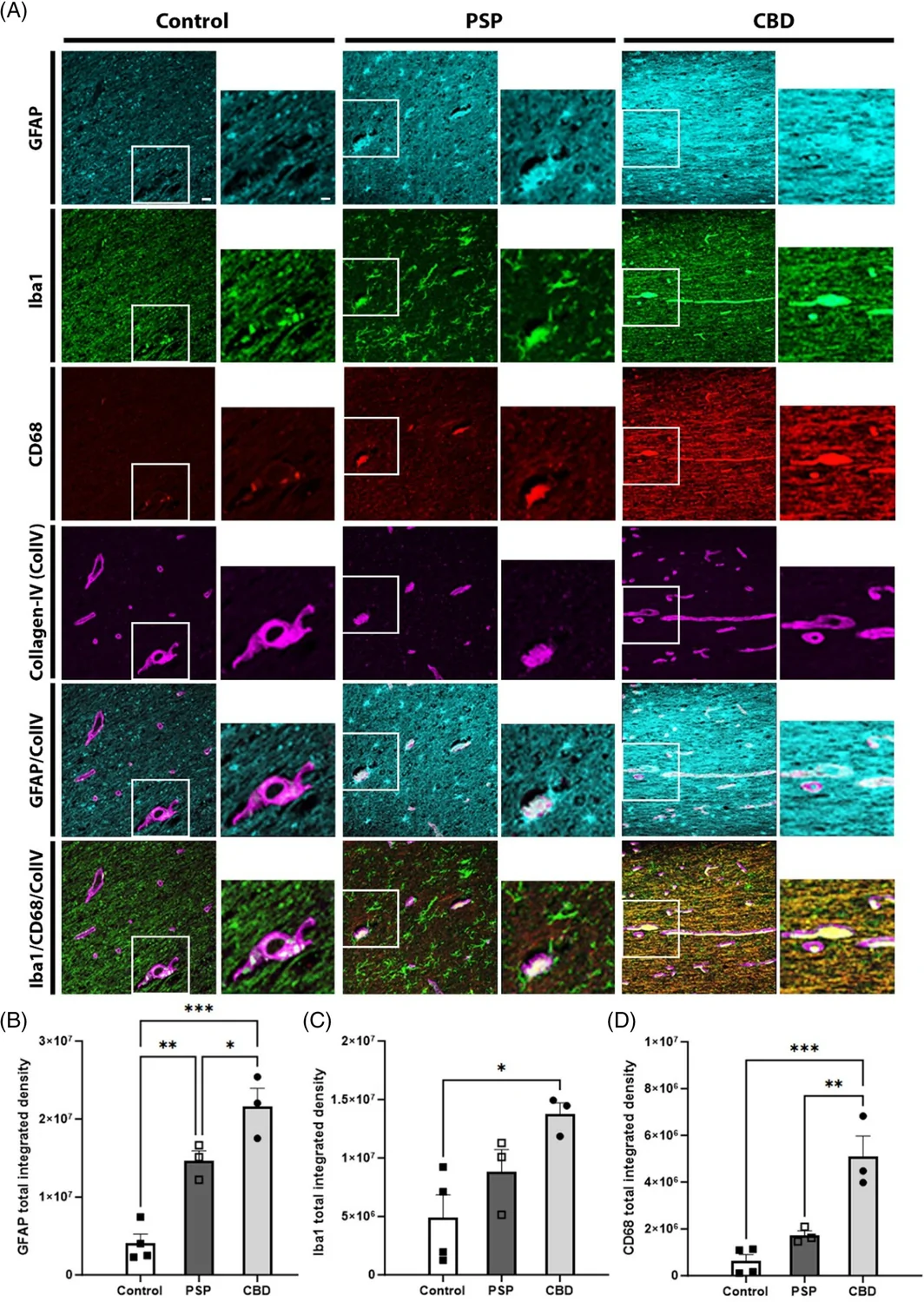
(A) Immunofluorescent staining of white matter (WM) of superior/middle frontal gyrus (S/M‐FG) of Controls, PSP and CBD. Each row represents immunofluorescent labeling with GFAP (cyan), Iba1 (green), CD68 (red), and Collagen IV (ColIV‐magenta) and merged images (GFAP/ColIV, Iba1/CD68/ColIV). White rectangles show where inserts are taken and placed to the right side of each image. Scale bars = 10 and 20 μm, respectively. Total integrated density of (B) GFAP, (C) Iba1, and (D) CD68 immunoreactive area around vasculature are shown. Controls; *n* = 4, PSP and CBD; *n* = 3 cases. * *p*< 0.05, **p< 0.01, ***p< 0.001, One‐way ANOVA and Tukey post‐hoc multiple pairwise comparisons.

**FIGURE 2:**
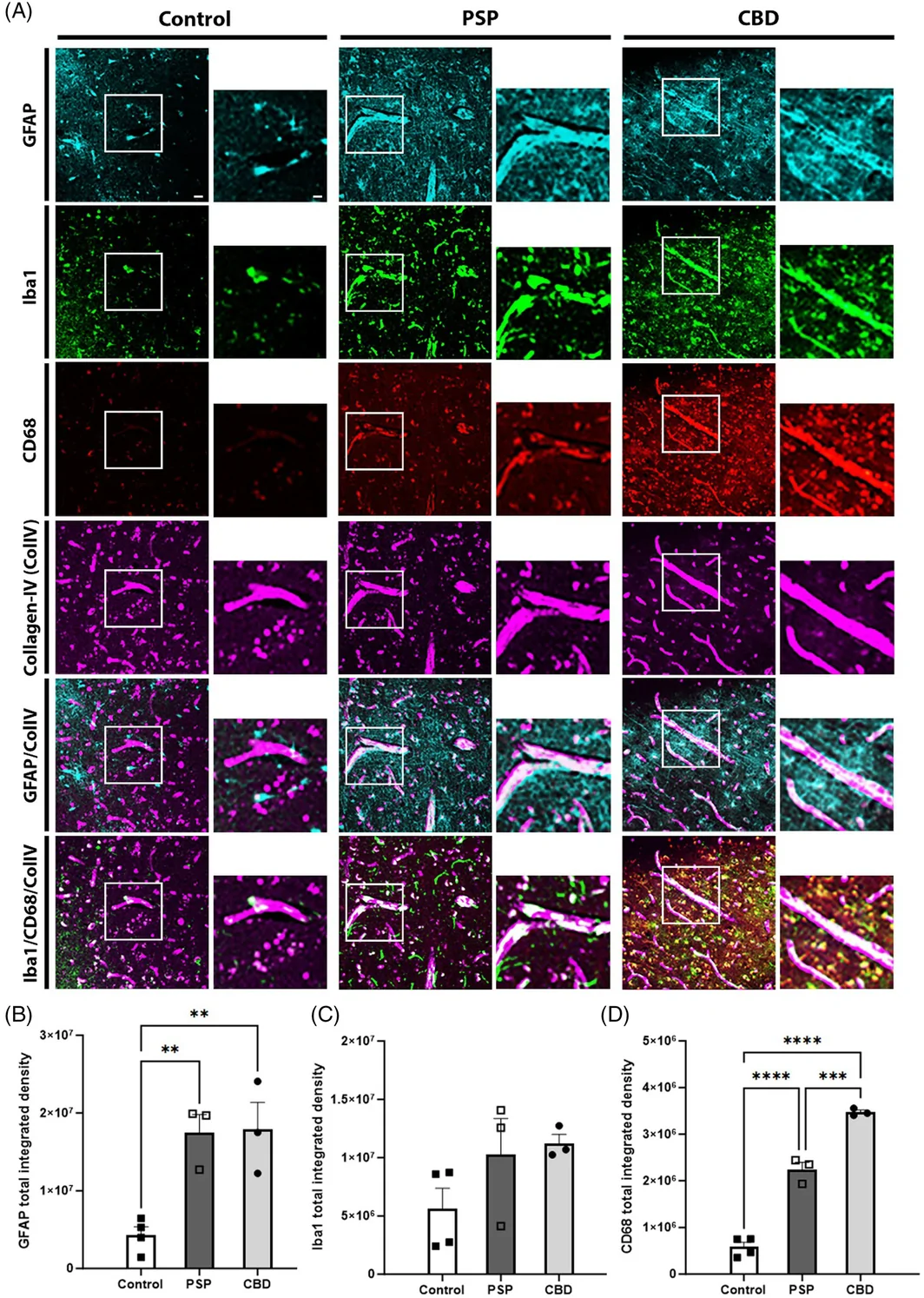
(A) Immunofluorescent staining of gray matter (GM) of superior/middle frontal gyrus (S/M‐FG) of Controls, PSP and CBD. Each row represents immunofluorescent labeling with GFAP (cyan), Iba1 (green), CD68 (red), and Collagen IV (ColIV‐magenta) and merged images (GFAP/ColIV, Iba1/CD68/ColIV). White rectangles show where inserts are taken and placed in the right column of each image. Scale bars = 10 and 20 μm, respectively. Total integrated density of (B) GFAP, (C) Iba1, and (D) CD68 immunoreactive area around vasculature are shown. Controls; *n* = 4, PSP and CBD; *n* = 3 cases. **p< 0.01, ***p< 0.001, ****p< 0.0001. One‐way ANOVA and Tukey post‐hoc multiple pairwise comparisons.

**FIGURE 3:**
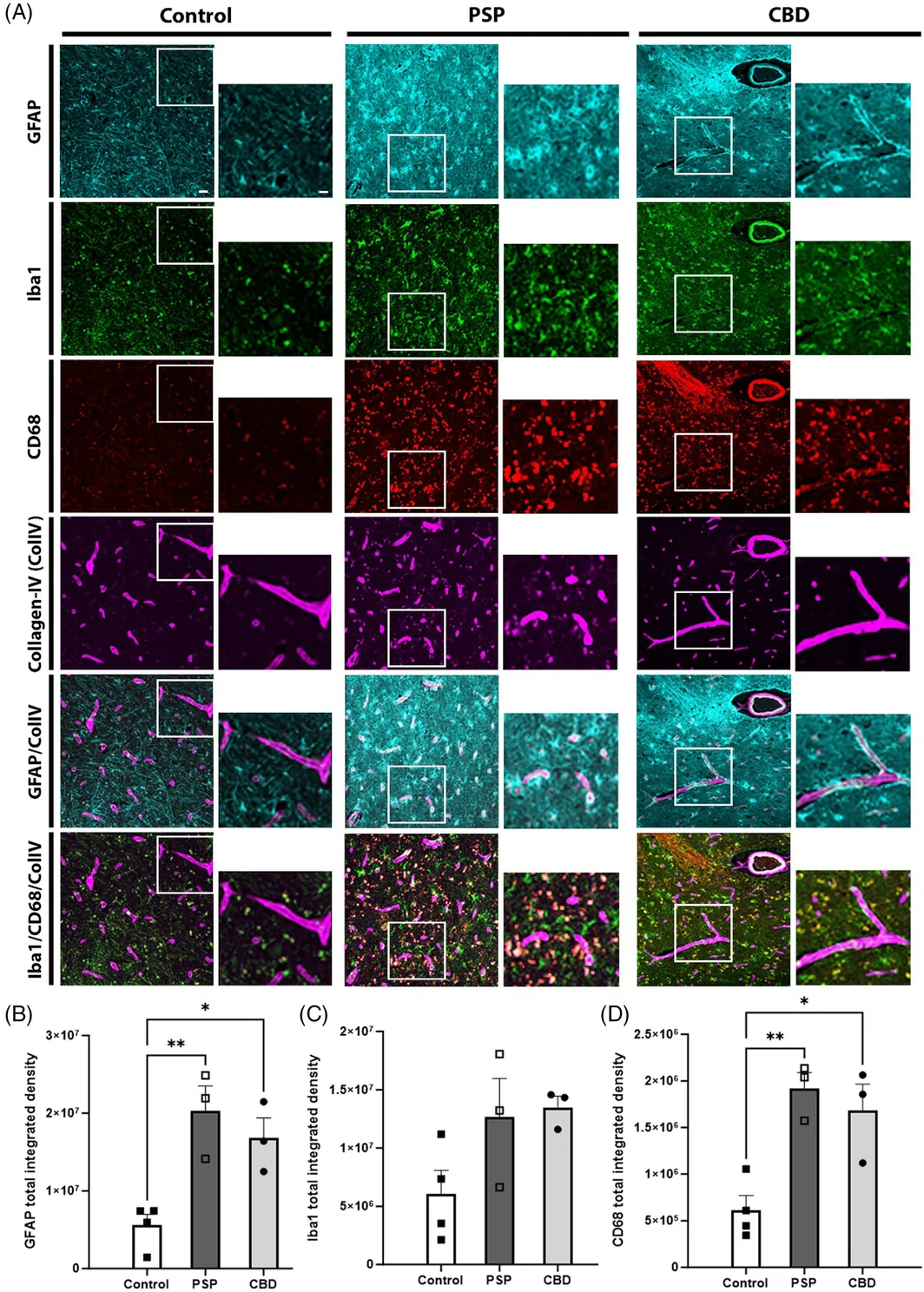
(A) Immunofluorescent staining of basal ganglia (Globus pallidus‐internus (GPi)) of Controls, PSP and CBD. Each row represents immunofluorescent labeling with GFAP (cyan), Iba1 (green), CD68 (red), and Collagen IV (ColIV‐magenta) and merged images (GFAP/ColIV, Iba1/CD68/ColIV). White rectangles show where inserts are taken and placed in the right column of each image. Scale bars = 10 and 20 μm, respectively. Total integrated density of (B) GFAP, (C) Iba1, and (D) CD68 immunoreactive area around vasculature are shown. Controls; *n* = 4, PSP and CBD; *n* = 3 cases. * *p*< 0.05, **p< 0.01. One‐way ANOVA and Tukey post‐hoc multiple pairwise comparisons.

### 3.3 Peri‐venular and arteriolar proinflammatory activation in PSP and CBD

To further elucidate the histopathological relationship between vessel types and proinflammatory activation, we labeled venules and arterioles with MCT1 and αSMA [43] along with GFAP which was previously shown as the best marker to assess chronic sustained proinflammatory activation [31]. Our results showed that both peri‐arteriolar and peri‐venular proinflammatory activation, assessed via quantifying perivascular GFAP+ astrocytes, in CBD was greater than PSP and controls in both the WM (Figure 4) and GM of the S/M‐FG (Figure 5). The relative GFAP+ burden (vein/artery %) in the WM was almost 3‐fold greater in PSP than controls, reflecting greater involvement of venules, whereas CBD affected both vessel types similarly (Figure 4A–D). However, the relative GFAP+ burden did not differ between groups in the GM, demonstrating that both arterioles and venules were affected similarly in PSP and CBD (Figure 5A–D). The relative GFAP+ burden was greater in PSP compared to controls and CBD in the GPi, GPe and Put (Figures 6, S5, and S6). Like the S/M‐FG, the peri‐arteriolar and peri‐venular GFAP+ density was greater in CBD than controls in all BG regions (Figures 6, S5, and S6). However, PSP also showed greater peri‐venular GFAP+ density in all BG regions (Figures 6, S5, and S6).

**FIGURE 4:**
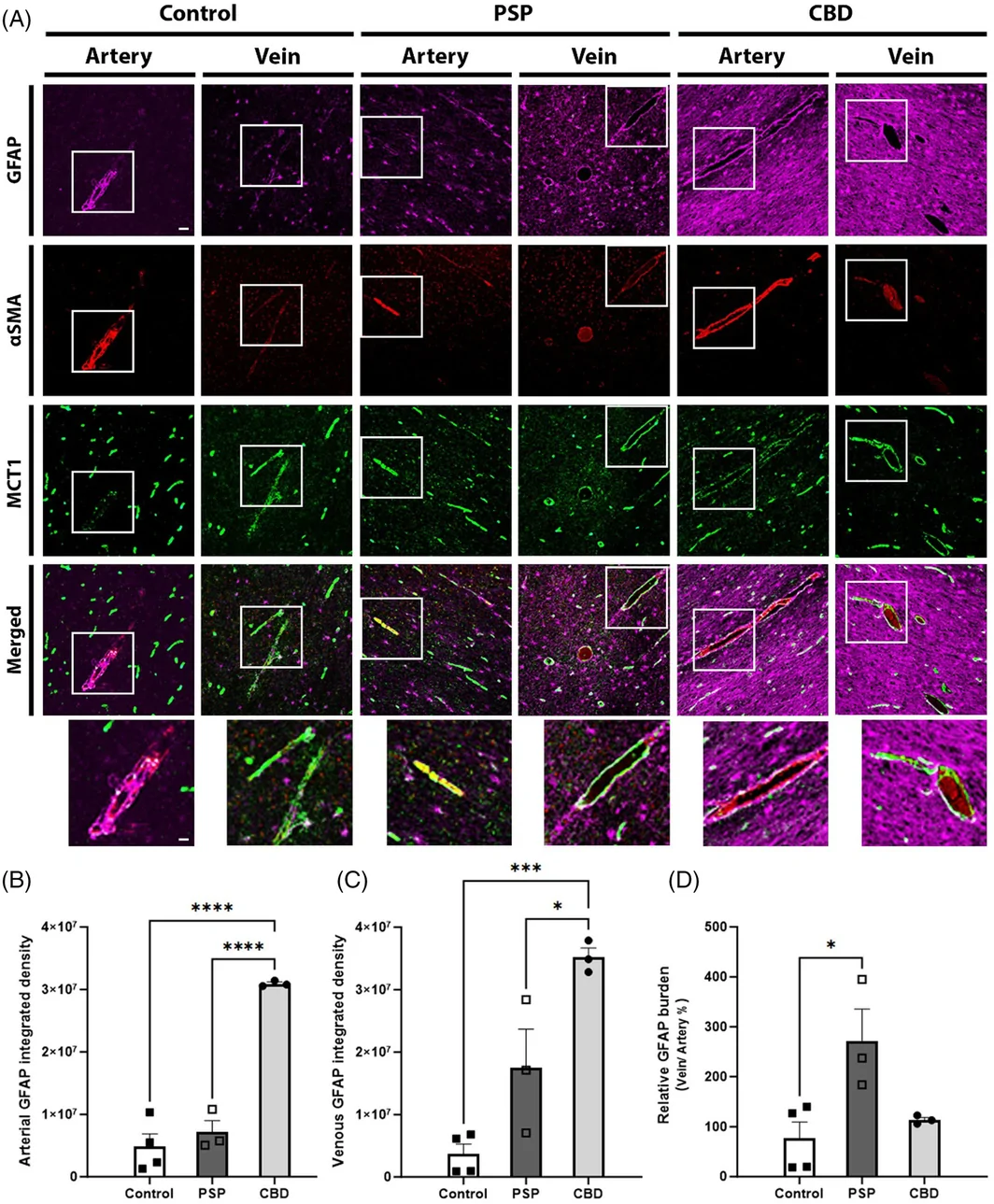
(A) Immunofluorescent staining of white matter (WM) of superior/middle frontal gyrus (S/M‐FG) of Controls, PSP and CBD. Each row represents immunofluorescent labeling with GFAP (magenta), alpha‐smooth muscle Actin (*α*SMA) (red), monocarboxylate transporter‐1 (MCT1) (green), and merged. Photomicrographs taken in arteriolar and venular territories from each case are shown in separate columns. White rectangles show where inserts are taken and placed in the bottom row in each territory. Scale bar = 10 μm. Total integrated density of GFAP immunoreactive area around (B) arteriolar, and (C) venular territories are shown. (D) Relative GFAP burden (vein/artery %) demonstrates the relative peri‐arteriolar and peri‐venular neuroinflammatory activation. Controls; *n* = 4, PSP and CBD; *n* = 3 cases. * *p*< 0.05, **p< 0.01, ***p< 0.001, ****p< 0.0001. One‐way ANOVA and Tukey post‐hoc multiple pairwise comparisons.

**FIGURE 5:**
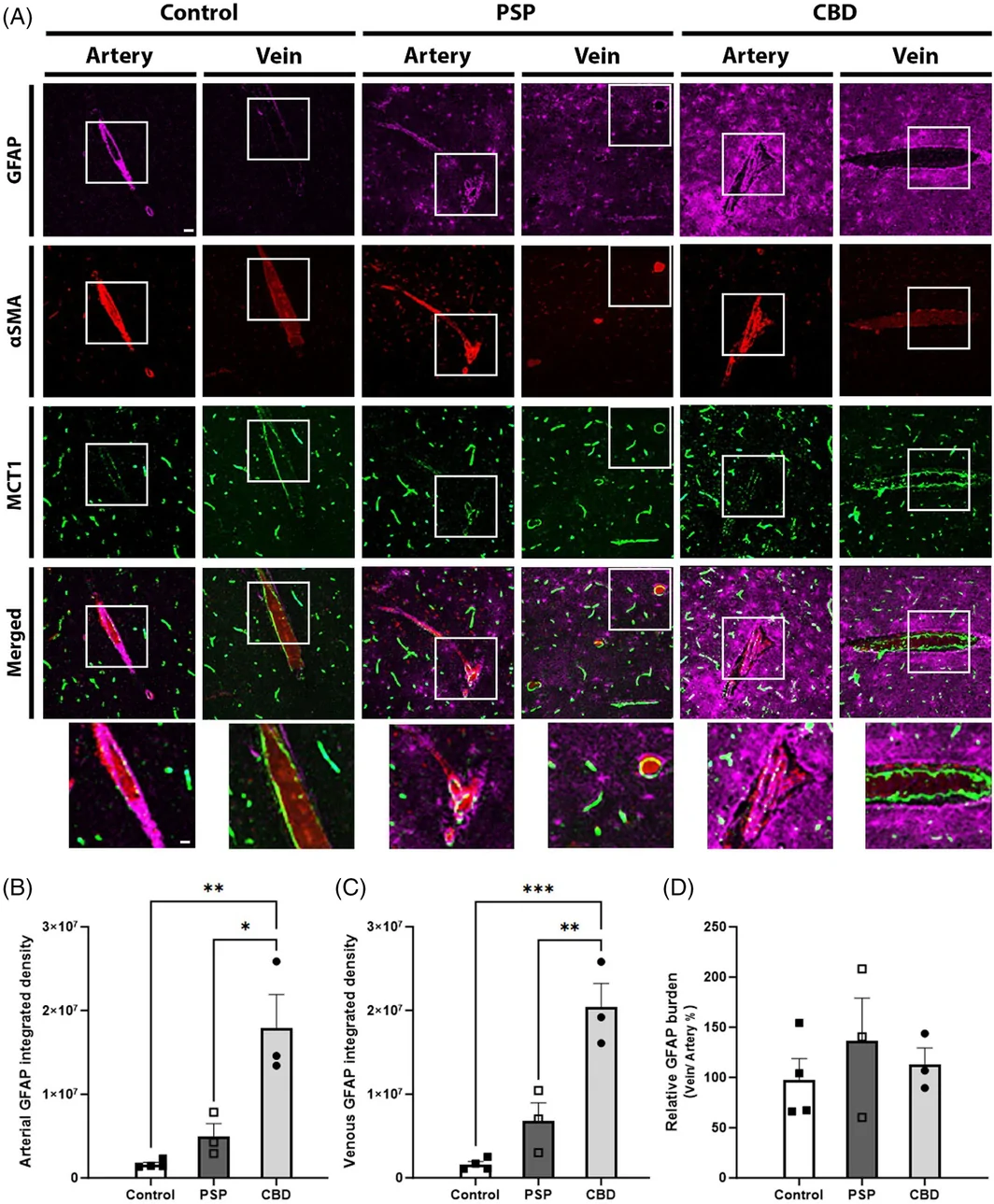
(A) Immunofluorescent staining of gray matter (GM) of superior/middle frontal gyrus (S/M‐FG) of Controls, PSP and CBD. Each row represents immunofluorescent labeling with GFAP (magenta), alpha‐smooth muscle Actin (αSMA) (red), monocarboxylate transporter‐1 (MCT1) (green), and merged. Photomicrographs taken in arteriolar and venular territories from each case are shown in separate columns. White rectangles show where inserts are taken and placed in the bottom row in each territory. Scale bar = 10 μm. Total integrated density of GFAP immunoreactive area around (B) arteriolar, and (C) venular territories are shown. (D) Relative GFAP burden (vein/artery %) demonstrates relative peri‐arteriolar and peri‐venular neuroinflammatory activation. Controls; *n* = 4, PSP and CBD; *n* = 3 cases. * *p* < 0.05, **p< 0.01, ***p< 0.001. One‐way ANOVA and Tukey post‐hoc multiple pairwise comparisons.

**FIGURE 6:**
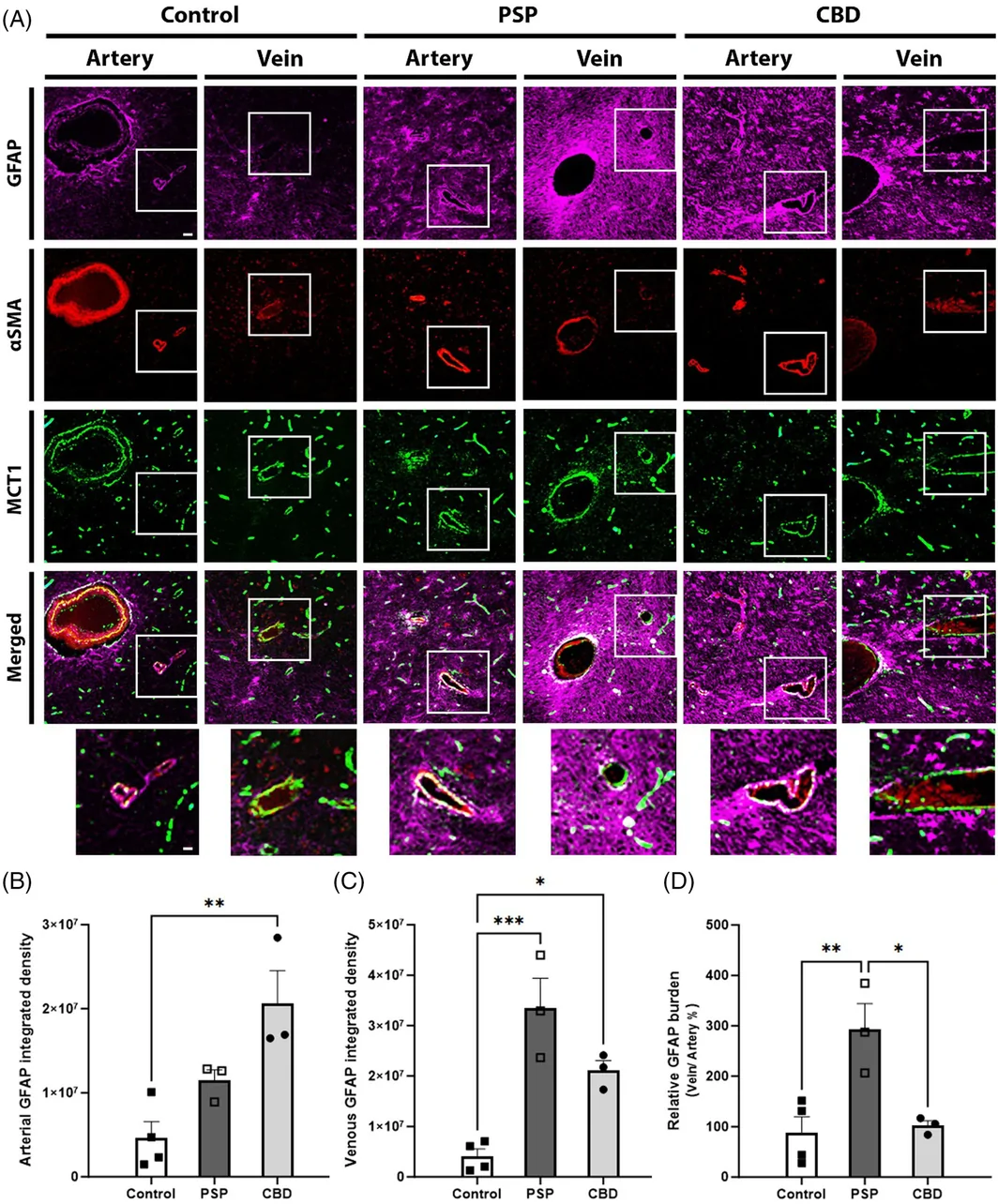
(A) Immunofluorescent staining of basal ganglia (Globus pallidus‐internus (GPi)) of Controls, PSP and CBD. Each row represents immunofluorescent labeling with GFAP (magenta), alpha‐smooth muscle Actin (αSMA) (red), monocarboxylate transporter‐1 (MCT1) (green), and merged. Photomicrographs taken in arteriolar and venular territories from each case are shown in separate columns. White rectangles show where inserts are taken and placed in the bottom row in each territory. Scale bar = 10 μm. Total integrated density of GFAP immunoreactive area around (B) arteriolar, and (C) venular territories are shown. (D) Relative GFAP burden (vein/artery %) demonstrates relative peri‐arteriolar and peri‐venular neuroinflammatory activation. Controls; *n* = 4, PSP and CBD; *n* = 3 cases. * *p*< 0.05, **p< 0.01, ***p< 0.001. One‐way ANOVA and Tukey post‐hoc multiple pairwise comparisons.

### 3.4 Peri‐vascular tau and proinflammation in PSP and CBD

We also assessed whether the proinflammatory activation was directly linked to the accumulation of tau within perivascular or vessel wall. AT8 immunolabeling, the gold standard phosphorylated tau marker, along with ColIV and GFAP/Iba1, was stained in S/M‐FG WM (Figure S7) and GM (Figure S8). The findings demonstrated virtually no colocalization of tau with vascular structures in PSP and CBD cases.

## 4 DISCUSSION

This study is the first to elucidate the neuroinflammatory changes around different types of vasculatures in 4R‐tauopathies. The proinflammatory changes in CBD were prominent and widespread in the WM and GM of the S/M‐FG, and the BG, and affected both arterioles and venules. In contrast, the major perivascular proinflammatory changes in PSP were more limited to the BG and preferentially affected post‐capillary venules.

### 4.1 Neuroinflammation and 4R‐tauopathies

In our studies, we identified astrocytes and resident microglia/infiltrating macrophages (microglia) by using cellular inflammatory markers GFAP, Iba1, and CD68. A striking feature of CBD was the WM of the S/M‐FG being occupied with highly expressed GFAP astrogliosis and abundant Iba1^+^ microglia with increased CD68 expression; meanwhile, the proinflammatory activation in PSP was more limited to the BG. This pattern coincided with the regional distribution of tau (AT8) in these 4R‐tauopathies [45]. The reciprocal augmentation between tau and neuroinflammation has been proved both in vitro and in vivo [46, 47]. Aside from consistency with the neuropathological picture, the perivascular proinflammatory pattern in our study corresponded to the microglial activation region of PSP and CBD patients based on the [^11^C]PK11195 PET studies [48, 49]. Using both [^11^C]PK11195 and [^18^F]AV‐1451 PET, the extent of neuroinflammation was correlated with the clinical manifestation and disease progression of AD and PSP [50, 51]. The intimate relationship between neuroinflammation and tau in PSP and CBD suggests a potential pathophysiological role of inflammation in these 4R‐tauopathies and implies that anti‐inflammation as a new therapeutic strategy might be worth studying.

Among BG structures, GPi showed greater inflammation than the others and this result is in accordance with the pathological and [^18^F]PI‐2620 tau‐PET studies [52, 53]. The venous system responsible for draining BG is the deep cerebral veins, and internal cerebral veins (ICVs) are the key structures in this system [54]. ICVs are located within the third ventricle, and regions closer to the ventricle would be more affected if there is a drainage problem in ICVs. Besides, GPi blood flow is supplied by the most distal end of lenticulostriate arteries which might make this region more susceptible to the perivascular proinflammatory activation [55]. Further studies are needed to confirm whether there is deep cerebral venous insufficiency or lenticulostriate arteriopathy in the 4R‐tauopathies. In the frontal gyrus, the inflammation was more prominent in the WM than GM. Compared with GM, the territory of WM is broader, and the draining veins are larger. During BBB disturbance, the recruitment of immune cells mainly occurs at postcapillary venules [56]. Furthermore, penetrating arteries come to their distal end in WM and cause hemodynamic susceptibility in this region. These vascular characteristics might explain the differential perivascular proinflammatory activation in the white and gray matter of S/M‐FG [57, 58].

Three proinflammatory markers were used in our studies, including Iba1, CD68, and GFAP. After careful examination of the correlations with proinflammatory changes, GFAP was found to be the most consistent marker across our studies, a finding that echoes the observations in AD [31]. Iba1 and CD68 are important markers identifying microglia/macrophage activation, while GFAP is an index of astrogliosis. Our findings suggest that astrogliosis represents a key pathological feature of 4R‐tauopathies and should be included in the further histopathological assessments [29, 59].

### 4.2 Cerebral vasculature and neuroinflammation

The preferential distribution of perivascular inflammation‐ pan‐vascular type in CBD and subcortical venules in PSP, implies that these 4R‐tauopathies affect unique aspects of cerebral blood flow (CBF) systems. The S/M‐FG is a cortical region, where CBF depends on the penetrating arterioles from pial arteries and superficial cortical venules to pial veins [60]. For BG, the arterial supply and venous drainage rely on the deep perforating arterioles and deep medullary venules [61]. Postcapillary venules are specialized sites, the endothelial cells in these regions do not have stress fibers and fluid is reuptaken from the interstitium. The lower shearing stress and higher permeability render them more susceptible to leukocyte adhesion and inflammatory responses [62, 63]. In addition, the cerebral venous system is valveless allowing bidirectional flow, thus facilitating the spread of pathogenic conditions [64, 65]. Lesions in the arterial system cause blocked inflow and tissue ischemia, while dysfunction of venular system causes outflow obstruction and vascular edema [66]. Vascular endothelium is the core component of BBB, and dysfunction of BBB leads to further cellular and architectural damage in the corresponding brain region [67]. Taken together, the pan‐vasculitis in CBD patients may contribute to the plethora of symptoms encompassing cortical and subcortical manifestations [68]. The preferential venular involvement of the PSP patients not only explains the subcortical symptoms [9], but also support that the pathophysiology of the increased WMHs within deep venous system might attribute to venous congestion [40]. In the puzzled PSP‐CBS clinical syndrome, whether there is dispersion of inflammation between the superficial and deep venous system contributing to the varied clinical manifestation remains undetermined and requires further investigation.

Perivascular inflammation encompasses a complex interplay among activated astrocytes, resident microglia, infiltrating macrophages and other inflammatory cells [56]. The proinflammatory activation of astrocytes and microglia/macrophages is evident everywhere; however, vessel types exhibited distinct influences on the CNS inflammation in our findings. The glymphatic system is a brain‐wide network of perivascular pathways, involving both artery and vein, subserving the clearance of waste products in the CNS [69]. Alongside the glymphatic system, the burgeoning field of meningeal lymphatic vessels (mLVs) ties closely to neurodegenerative diseases [70, 71, 72]. The major mLVs were detected in the superior sagittal sinus (SSS) and falx cerebri [73, 74], and surprisingly these regions are in close proximity to the characteristic atrophic regions of CBD [75]. The coincidence of regional proximity and the distinguishing perivascular proinflammatory responses in our work supports the close crosstalk between CBF dynamics and inflammation. Although our results suggest that the impairment of waste clearance in 4R‐tauopathies might be related to inflammation, tracing the inflammatory origin of PSP and CBD is beyond the scope of this study.

### 4.3 Vasculopathies and 4R‐tauopathies

Here, we meticulously dissected vasculatures in different regions of PSP and CBD from the perspective of neuroinflammation. In our results, the differential distribution of perivascular proinflammation in the preferential vasculature coincides with the characteristics of tau distribution in both 4R‐tauopathies [45]. However, tau has not been demonstrated to localize in the perivascular space or in the vessel wall. Whether this association arises primarily from their anatomical proximity or is mediated by other pathological processes warrants further investigation. Mounting evidence demonstrated that iron preferentially co‐localize with tau or accumulate in its vicinity [76, 77]. When there is endothelial injury during pathological condition, hemoglobin is released and breakes down into heme and redox‐active iron, creating oxidative stress and accelerating vascular injury [78]. Althogh the exact interaction between tau, vasculopathy, and iron remains unknown, vasculopathy may be an important factor in the development of 4R‐tauopathies based on our study.

### 4.4 Strengths and limitations

This is the first study disclosing vasculopathy and proinflammatory activation pertaining to different vessel types in 4R‐tauopathies. The selected cases were neuropathologically confirmed homogeneous representative cases. We employed multiplex immunofluorescence to reveal the spatial relationship among vasculature and proinflammatory activation in microglia and astrocytes. Thin sections hinder getting the whole picture of the larger vascular network though we tried to span more images from each region per case. Besides, cytokine release and transcriptome were not analyzed to fully support the proinflammatory activation due to technical difficulties. The relatively small sample size may have limited the generalizability of our findings, and larger independent cohorts are warranted for validation.

## 5 CONCLUSIONS

Perivascular inflammatory changes are common features in both PSP and CBD, particularly in CBD. The pan‐vascular involvement in CBD and the venule‐predominant inflammatory phenotype in PSP correspond to the affected brain regions and the clinical manifestations of these 4R‐tauopathies. Our findings implicate that vasculopathies are central to the pathophysiology of these neurodegenerative diseases. Future studies are needed to better elucidate the crosstalk among inflammation, vascular system, and tau proteinopathies to achieve a more comprehensive understanding of disease mechanisms.

## AUTHOR CONTRIBUTIONS

**Mu‐Hui Fu:** Writing—original draft; methodology; investigation; conceptualization. **Gokhan Uruk:** Writing—original draft; methodology; investigation; formal analysis. **Rodolfo G. Gatto:** Methodology; formal analysis; investigation. **R. Ross Reichard:** Resources; project administration; investigation. **Jennifer L. Whitwell:** Writing—review and editing; project administration; supervision; funding acquisition. **Keith A. Josephs:** Writing—review and editing; project administration; supervision; funding acquisition.

## FUNDING INFORMATION

This study was funded by National Institutes of Health (NIH) grants R01‐NS89757, R01‐AG87140, and RF1‐NS112153.

## CONFLICT OF INTEREST STATEMENT

The authors report no competing interests.

## Supporting information


**Table S1.** Demographics and Neuropathological diagnosis of subjects of study.
**Figure S1.** Representative MRI from control and neuropathologically confirmed PSP and CBD cases. (A) Axial MRI FLAIR view at the level of the cortical regions, centered on the superior (S‐MG) and middle (M‐FG) frontal gyrus subregions. Regions of interest (white squares) show a hyperintense signal in the white matter (WM) (indicated by lines and arrow) in subjects with neuropathologically confirmed PSP and CBD. (B) Axial MRI FLAIR view centered on the putamen (Put) and globus pallidus (GP) within the subcortical basal ganglia (BG) region. Note the inhomogeneities in the BG regions of the PSP subject (arrows).
**Figure S2.** (A) Immunofluorescent staining of basal ganglia (Globus pallidus‐externus (GPe)) of Controls, PSP and CBD. Each row represents immunofluorescent labeling with GFAP (cyan), Iba1 (green), CD68 (red), and Collagen IV (ColIV‐magenta) and merged images (GFAP/ColIV, Iba1/CD68/ColIV). White rectangles show where inserts are taken and placed in the right column of each image. Scale bars = 10 and 20 μm, respectively. Total integrated density of (B) GFAP, (C) Iba1, and (D) CD68 immunoreactive area around vasculature are shown. Controls; *n* = 4, PSP and CBD; *n* = 3 cases. * *p*< 0.05, ** *p*< 0.01. One‐way ANOVA and Tukey post‐hoc multiple pairwise comparisons.
**Figure S3.** (A) Immunofluorescent staining of basal ganglia (putamen) of Controls, PSP and CBD. Each row represents immunofluorescent labeling with GFAP (cyan), Iba1 (green), CD68 (red), and Collagen IV (ColIV‐magenta) and merged images (GFAP/ColIV, Iba1/CD68/ColIV). White rectangles show where inserts are taken and placed in the right column of each image. Scale bars = 10 and 20 μm, respectively. Total integrated density of (B) GFAP, (C) Iba1, and (D) CD68 immunoreactive area around vasculature are shown. Controls; *n* = 4, PSP and CBD; *n* = 3 cases. *** *p*< 0.001. One‐way ANOVA and Tukey post‐hoc multiple pairwise comparisons.
**Figure S4.** Related to Figures 1, 2, 3, S2, S3. Comparsion among S/M‐FG, and BG subregions of total integrated density of (A)–(D) GFAP, (E)–(H) Iba1, and (I)–(L) CD68 immunoreactive area around vasculature are shown. Controls; *n* = 4, PSP and CBD; *n* = 3 cases. ns = non‐significant. One‐way ANOVA and Tukey post‐hoc multiple pairwise comparisons.
**Figure S5.** (A) Immunofluorescent staining of basal ganglia (Globus pallidus‐externus (GPe)) of Controls, PSP and CBD. Each row represents immunofluorescent labeling with GFAP (magenta), alpha‐smooth muscle actin (*α*SMA) (red), monocarboxylate transporter‐1 (MCT1) (green), and merged. Photomicrographs taken in arteriolar and venular territories from each case are shown in separate columns. White rectangles show where inserts are taken and placed in the bottom row in each territory. Scale bar = 10 μm. Total integrated density of GFAP immunoreactive area around (B) arteriolar, and (C) venular territories are shown. (D) Relative GFAP burden (vein/artery %) demonstrates relative peri‐arteriolar and peri‐venular neuroinflammatory activation. Controls; *n* = 4, PSP and CBD; *n* = 3 cases. * *p*< 0.05, ** *p*< 0.01, *** *p*< 0.001. One‐way ANOVA and Tukey post‐hoc multiple pairwise comparisons.
**Figure S6.** (A) Immunofluorescent staining of basal ganglia (putamen) of Controls, PSP and CBD. Each row represents immunofluorescent labeling with GFAP (magenta), alpha‐smooth muscle actin (*α*SMA) (red), monocarboxylate transporter‐1 (MCT1) (green), and merged. Photomicrographs taken in arteriolar and venular territories from each case are shown in separate columns. White rectangles show where inserts are taken and placed in the bottom row in each territory. Scale bar = 10 μm. Total integrated density of GFAP immunoreactive area around (B) arteriolar, and (C) venular territories are shown. (D) Relative GFAP burden (vein/artery %) demonstrates relative peri‐arteriolar and peri‐venular neuroinflammatory activation. Controls; *n* = 4, PSP and CBD; *n* = 3 cases. * *p*< 0.05, ** *p*< 0.01, *** *p*< 0.001. One‐way ANOVA and Tukey post‐hoc multiple pairwise comparisons.
**Figure S7.** (A) Immunofluorescent staining of white matter (WM) of superior/middle frontal gyrus (S/M‐FG) of Controls, PSP and CBD. Each row represents immunofluorescent labeling with GFAP or Iba1 (red), Collagen IV (magenta), AT8+ tau (green), and merged. White rectangles show where inserts are taken and placed in the bottom row in each territory. Scale bars = 10 μm.
**Figure S8.** (A) Immunofluorescent staining of gray matter (GM) of superior/middle frontal gyrus (S/M‐FG) of Controls, PSP and CBD. Each row represents immunofluorescent labeling with GFAP or Iba1 (red), Collagen IV (magenta), AT8+ tau (green), and merged. White rectangles show where inserts are taken and placed in the bottom row in each territory. Scale bars = 10 μm.

## Data Availability

The data that support the findings of this study are available on request from the corresponding author. The data are not publicly available due to privacy or ethical restrictions.
