# Supplementary material for: The characteristics of perivascular proinflammation in 4‐repeat tauopathies
Source: Brain Pathol. 2026 Oct 28:e70162. Online ahead of print. doi: 10.1111/bpa.70162 (PMC13716495; doi:10.1111/bpa.70162)
Supplement: Supplementary file 1 [file BPA-9999-e70162-s001.docx]

**The characteristics of perivascular proinflammation in 4-repeat tauopathies**

Mu-Hui Fu, MD, PhD^1,2*^; Gokhan Uruk, MD, PhD^1*^; Rodolfo G. Gatto, MD, PhD^1^; R. Ross Reichard, MD^3^; Jennifer L. Whitwell, PhD^4^; Keith A. Josephs, MD, MST, MSc^1†^

^1^Department of Neurology, Mayo Clinic, Rochester, MN, USA

^2^Department of Neurology, Kaohsiung Chang Gung Memorial Hospital and Chang Gung University College of Medicine, Kaohsiung 83301, Taiwan

^3^Department of Laboratory Medicine and Pathology (Neuropathology), Mayo Clinic, Rochester, MN, USA

^4^Department of Radiology, Mayo Clinic, Rochester, MN, USA

*Authors equally contributed to the manuscript

**Running title:** *Perivascular proinflammation in 4R-tauopathies*

Email address:

Mu-Hui Fu: [FU.MUHUI@mayo.edu](mailto:FU.MUHUI@mayo.edu)

Gokhan Uruk: [Uruk-Contreras.Gokhan@mayo.edu](mailto:Uruk-Contreras.Gokhan@mayo.edu)

Rodolfo G. Gatto: [Gatto.Rodolfo@mayo.edu](mailto:Gatto.Rodolfo@mayo.edu)

R. Ross Reichard: [Reichard.Robert@mayo.edu](mailto:Reichard.Robert@mayo.edu)

Jennifer L. Whitwell: [Whitwell.Jennifer@mayo.edu](mailto:Whitwell.Jennifer@mayo.edu)

Keith A. Josephs: [Josephs.Keith@mayo.edu](mailto:Josephs.Keith@mayo.edu)

Acknowledgment: We would like to thank all patients, families, and contributors who made this research study possible.

**^†^Correspondence author:**

Keith A. Josephs, MD, MST, MSc

Professor of Neurology and Neuroscience

Department of Neurology,

Mayo Clinic College of Medicine and Science

200 1^st^ St SW, Rochester, MN55905, USA

E-mail: [josephs.keith@mayo.edu](mailto:josephs.keith@mayo.edu)

**Supplementary Data**

**Table S1.**  **Demographics and Neuropathological diagnosis of subjects of study**

| **Case #** | **Clinical dx** | **Neuropath Primary dx** | **Neuropath Secondary dx** | **Sex** | **Age** | **Disease length (y)** | **PET to death (y)** | **ADNC** |
| --- | --- | --- | --- | --- | --- | --- | --- | --- |
| 1 | Control (Non-Tau) | none reported | none reported | M | 84 | - | NA | A0B0C0 |
| 2 | Control (Non-Tau) | none reported | none reported | M | 67 | - | NA | A0B0C0 |
| 3 | Control (Non-Tau) | none reported | none reported | F | 80 | - | NA | A0B0C0 |
| 4 | Control (Non-Tau) | none reported | none reported | M | 55 | - | NA | A0B0C0 |
| 5 | PSP-RS | PSP | none reported | M | 65 | 5 | 1 | A0B1C0 |
| 6 | PSP-SL | PSP | none reported | F | 83 | 6 | 2 | A0B1C0 |
| 7 | PSP-F | PSP | CVD | M | 74 | 5 | NA | A0B0C0 |
| 8 | FTD | CBD | none reported | F | 75 | 2 | 2 | A0B1C0 |
| 9 | FTD | CBD | TDP-43, ARTAG | F | 67 | 8 | 3 | A0B1C0 |
| 10 | FTD | CBD | TDP-43, ARTAG, AGD | F | 73 | 3 | 1 | A0B1C0 |

M, male; F, female; ADNC, AD neuropathological changes; AGD, argyrophilic grain disease; ARTAG, Age-related tau astrogliopathy; CBD, corticobasal degeneration; CVD, cerebrovascular disease; dx, diagnosis; FTD, frontotemporal dementia; NA, not available; PSP, progressive supranuclear palsy; TDP-43, TAR DNA-binding protein 43.

**Figure S1.** Representative MRI from control and neuropathologically confirmed PSP and CBD cases. (A) Axial MRI FLAIR view at the level of the cortical regions, centered on the superior (S-MG) and middle (M-FG) frontal gyrus subregions. Regions of interest (white squares) show a hyperintense signal in the white matter (WM) (indicated by lines and arrow) in subjects with neuropathologically confirmed PSP and CBD. (B) Axial MRI FLAIR view centered on the putamen (Put) and globus pallidus (GP) within the subcortical basal ganglia (BG) region. Note the inhomogeneities in the BG regions of the PSP subject (arrows).

**Figure S2.** (A) Immunofluorescent staining of basal ganglia (Globus pallidus-externus (GPe)) of Controls, PSP and CBD. Each row represents immunofluorescent labeling with GFAP (cyan), Iba1 (green), CD68 (red), and Collagen IV (ColIV-magenta) and merged images (GFAP/ColIV, Iba1/CD68/ColIV). White rectangles show where inserts are taken and placed in the right column of each image. Scale bars = 10 and 20 µm, respectively. Total integrated density of (B) GFAP, (C) Iba1, and (D) CD68 immunoreactive area around vasculature are shown. Controls; n=4, PSP and CBD; n=3 cases. * p<0.05, ** p<0.01. One-way ANOVA and Tukey post-hoc multiple pairwise comparisons.

**Figure S3.** (A) Immunofluorescent staining of basal ganglia (putamen) of Controls, PSP and CBD. Each row represents immunofluorescent labeling with GFAP (cyan), Iba1 (green), CD68 (red), and Collagen IV (ColIV-magenta) and merged images (GFAP/ColIV, Iba1/CD68/ColIV). White rectangles show where inserts are taken and placed in the right column of each image. Scale bars = 10 and 20 µm, respectively. Total integrated density of (B) GFAP, (C) Iba1, and (D) CD68 immunoreactive area around vasculature are shown. Controls; n=4, PSP and CBD; n=3 cases. *** p<0.001. One-way ANOVA and Tukey post-hoc multiple pairwise comparisons.

**Figure S4.** Related to Figure 1,2,3, S2, S3. Comparsion among S/M-FG, and BG subregions of total integrated density of (A-D) GFAP, (E-H) Iba1, and (I-L) CD68 immunoreactive area around vasculature are shown. Controls; n=4, PSP and CBD; n=3 cases. ns= non-significant. One-way ANOVA and Tukey post-hoc multiple pairwise comparisons.

**Figure S5.** (A) Immunofluorescent staining of basal ganglia (Globus pallidus-externus (GPe)) of Controls, PSP and CBD. Each row represents immunofluorescent labeling with GFAP (magenta), alpha-smooth muscle actin (αSMA) (red), monocarboxylate transporter-1 (MCT1) (green), and merged. Photomicrographs taken in arteriolar and venular territories from each case are shown in separate columns. White rectangles show where inserts are taken and placed in the bottom row in each territory. Scale bar = 10 µm. Total integrated density of GFAP immunoreactive area around (B) arteriolar, and (C) venular territories are shown. (D) Relative GFAP burden (vein/artery %) demonstrates relative peri-arteriolar and peri-venular neuroinflammatory activation. Controls; n=4, PSP and CBD; n=3 cases. * p<0.05, ** p<0.01, *** p<0.001. One-way ANOVA and Tukey post-hoc multiple pairwise comparisons.

**Figure S6.** (A) Immunofluorescent staining of basal ganglia (putamen) of Controls, PSP and CBD. Each row represents immunofluorescent labeling with GFAP (magenta), alpha-smooth muscle actin (αSMA) (red), monocarboxylate transporter-1 (MCT1) (green), and merged. Photomicrographs taken in arteriolar and venular territories from each case are shown in separate columns. White rectangles show where inserts are taken and placed in the bottom row in each territory. Scale bar = 10 µm. Total integrated density of GFAP immunoreactive area around (B) arteriolar, and (C) venular territories are shown. (D) Relative GFAP burden (vein/artery %) demonstrates relative peri-arteriolar and peri-venular neuroinflammatory activation. Controls; n=4, PSP and CBD; n=3 cases. * p<0.05, ** p<0.01, *** p<0.001. One-way ANOVA and Tukey post-hoc multiple pairwise comparisons.

**Figure S7.** (A) Immunofluorescent staining of white matter (WM) of superior/middle frontal gyrus (S/M-FG) of Controls, PSP and CBD. Each row represents immunofluorescent labeling with GFAP or Iba1 (red), Collagen IV (magenta), AT8+ tau (green), and merged. White rectangles show where inserts are taken and placed in the bottom row in each territory. Scale bars = 10 µm.

**Figure S8.** (A) Immunofluorescent staining of gray matter (GM) of superior/middle frontal gyrus (S/M-FG) of Controls, PSP and CBD. Each row represents immunofluorescent labeling with GFAP or Iba1 (red), Collagen IV (magenta), AT8+ tau (green), and merged. White rectangles show where inserts are taken and placed in the bottom row in each territory. Scale bars = 10 µm.
